# FNAB cytology of extra-cranial metastasis of glioblastoma multiforme may resemble a lung primary: A diagnostic pitfall

**DOI:** 10.1186/1742-6413-2-9

**Published:** 2005-06-20

**Authors:** Mamatha Chivukula, HE Dincer, Julie A Biller, Hendrikus G Krouwer, Grant Simon, Vinod Shidham

**Affiliations:** 1 Departments of Pathology, Medical College of Wisconsin, Milwaukee, WI, USA; 2 Pulmonary and Critical Care Medicine, Medical College of Wisconsin, Milwaukee, WI, USA; 3 Neurology and Neurosurgery; Medical College of Wisconsin, Milwaukee, WI, USA

**Keywords:** Glioblastoma multiforme, Fine needle aspiration biopsy cytology, FNA, lung tumor

## Abstract

**Background:**

As extra-cranial metastasis of glioblastoma multiforme (GBM) is rare, it may create a diagnostic dilemma especially during interpretation of fine needle aspiration biopsy (FNAB) cytology.

**Case presentation:**

We present transbronchial FNAB findings in a 62-year-old smoker with lung mass clinically suspicious for a lung primary. The smears of transbronchial FNAB showed groups of cells with ill-defined cell margins and cytological features overlapping with poorly differentiated non-small cell carcinoma. The tumor cells demonstrated lack of immunoreactivity for cytokeratin, thyroid transcription factor-1, and usual neuroendocrine markers, synaptophysin and chromogranin in formalin-fixed cellblock sections. However, they were immunoreactive for the other neuroendocrine immunomarker, CD56, suggesting neural nature of the cells. Further scrutiny of clinical details revealed a history of GBM, 13 months status-post surgical excision with radiation therapy and systemic chemotherapy. The tumor recurred 7 months earlier and was debulked surgically and with intra-cranial chemotherapy. Additional evaluation of tumor cells for glial fibrillary acidic protein (GFAP) immunoreactivity with clinical details resulted in final interpretation of metastatic GBM.

**Conclusion:**

Lack of clinical history and immunophenotyping may lead to a diagnostic pitfall with possible misinterpretation of metastatic GBM as poorly differentiated non-small cell carcinoma of lung in a smoker.

## Background

Glioblastoma multiforme (GBM) represents extreme anaplasia in astrocytic tumors. Similar to other CNS tumors, the extra-cranial metastasis of GBM is rare and is usually observed after the tumor has infiltrated the dural veins, the cranium, the extra-cranial soft tissue, or more frequently after the tumor debulking surgery for a recurrent tumor [4].

A literature search revealed only a few case reports of extra-cranial metastasis of primary GBM to various organs such as spleen [5], skin [8], heart [7], bone [6], cervical lymph nodes [9,11] and lung [10,12-14]. In all these cases the metastases occurred after the resection of primary intra-cranial tumor with an average time interval of 10 months. Only one case of a spontaneous metastasis of primary GBM to lungs is reported [13]. The diagnosis in most cases was based on biopsy and immunophenotyping with glial fibrillary acidic protein (GFAP).

Since the metastasis of GBM is rare, cytopathological interpretation for its differentiation from other tumors may be challenging. Correct interpretation for origin of the tumor is important in rendering a proper clinical management. We report a case in which optimum clinical history and immunohistochemical evaluation played a crucial role in preventing a diagnostic pitfall of misinterpreting FNAB cytology of lung mass in a smoker.

## Case presentation

A 62-year-old male presented with shortness of breath for one week. He had a smoking history of 10 pack-years [1 pack per day, per year, for 10 years]. The X-ray chest revealed a large pleural effusion with suggestion of a mass in the right upper lobe of lung (RUL) with massive hilar lymphadenopathy. The left lung parenchyma was normal and the pulmonary vasculature was not congested. A computerized tomography scan of the chest confirmed a mass in RUL.

Clinically, the tumor was considered as lung primary and pulmonary consult was requested. Bronchoscopy of RUL revealed an irregular friable mucosa. A transbronchial FNAB of the RUL mass was performed with other evaluations.

The patient had previous history of GBM in the right occipital area 13 months ago for which he underwent initial surgical excision with radiation therapy and systemic chemotherapy. The tumor recurred after 6 months for which he underwent extensive debulking and placement of gliadel wafers (BNCU-impregnated wafers).

## Methods

Cytomorphological features on adequacy evaluation of transbronchial FNAB smears of the RUL mass were consistent with poorly differentiated non-small cell tumor. Additional material for cellblock was recommended for elective immunophenotyping of tumor cells.

Other evaluations included an endobronchial biopsy from RUL, bronchoalveolar lavage of anterior segment of RUL for cytology, and thoracocentesis of right pleural effusion for cytology.

## Results

### Cytopathological findings of transbronchial FNAB of the mass

**Figure 1:**
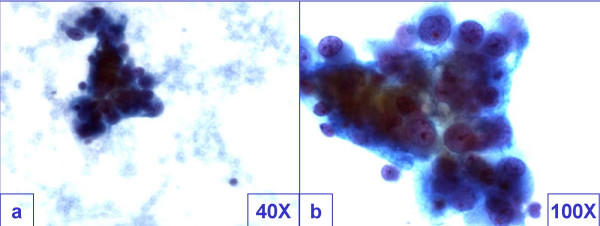
Cohesive groups of tumor cells showed mild pleomorphism. The cells had cyanophilic scant cytoplasm with indistinct cell borders with morphological features overlapping with poorly differentiated carcinoma.

The smears of the fine needle aspirates were relatively cellular with cohesive groups of tumor cells showing mild pleomorphism. The cells had cyanophilic scant cytoplasm with indistinct cell borders (Figure-1). The hyperchromatic nuclei showed powdery to clear chromatin with small but distinct nucleoli. Initial cytological differential diagnosis included poorly differentiated non-small cell carcinoma, paraganglioma, lymphoma, melanoma, and non-neoplastic lesion such as granuloma with addition of metastatic GBM after considering the history of surgical intervention for recurrent GBM.

**Figure 2:**
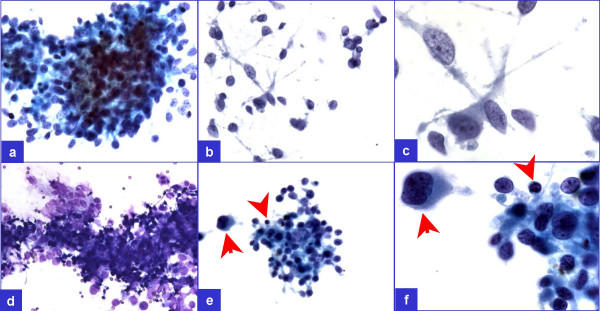
A few groups (a through d) did not show pleomorphism but showed occasional lymphocyte (arrowheads in e & f) admixed with the tumor cells demonstrating ill-defined cell borders superficially resembling a granuloma. However, pleomorphic cells (arrows in e & f) were not uncommon in this relatively cellular specimen.

As the smears were relatively cellular with significant proportion of tumor cell groups showing hyperchromasia and pleomorphism, granuloma could be excluded even with cytomorphological evaluation. However, a few groups without significant pleomorphism and without admixture with large tumor cells demonstrated overlapping morphological features with granuloma (Figure-2). In these groups, the cells showed ill-defined cytoplasm with slightly elongated, overlapping, oval nuclei. Occasional lymphocytes mixed with these cell groups were present, but multinucleated giant cells were absent.

Other malignant lesions in the differential diagnosis, except lymphoma, were relatively difficult to exclude by morphology alone. The cohesive tumor cells with clear to dusty chromatin and significant pleomorphism did not favor lymphoma. The nucleoli were relatively less prominent and did not favor melanoma and large cell carcinoma of lung. Paragaglioma could not be excluded morphologically except lack of Zellballen pattern (difficult to be evaluated cytologically) in the cellblock sections.

*Immunophenotyping *of tumor cells was performed on formalin-fixed paraffin-embedded cellblock sections (Table 1).

**Table 1:** Immnophenotype of tumor cells.

S.No.	Immunomarker	Antibody (Clone, source, dilution)	Antigen retrieval (Pre-treatment of sections)	Immunoreactivity pattern of tumor cells
1	Cytokeratin	Dako, 34BE12, 1:300 for 30 mts	Proteinase,-K for 4 mts	Non-immunoreactive
2	Cytokeratin 7	Dako, OVTL12/30, 1:100 for 30 mts	PH 6.0 at 98 C for 35 mts	Non-immunoreactive
3	Cytokeratin 20	Dako, Ks20.8, 1:300 for 30 mts	Proteinase, -K for 4 mts	Non-immunoreactive
4	TTF-1	Dako, 8G7G3/1, 1:150 for 20 mt	Heat DAKO, PH 6.0 for 35 mts	Non-immunoreactive
5	LCA	Dako, PD7/26/16 and 2B11, 1:200 for 30 mts	Proteinase, -K for 4 mts	Non-immunoreactive
6	MART-1	SIGNET, M2-7C10 1:600 for 30 mts	Heat, PH6.0 for 35 mts, 1:500 for 35 mts	Non-immunoreactive
7	Chromogranin	Novocastra, LK2H10, 1:600 for 30 mts	HeatPH 6.0 for 1:600 for 35 mts	Non-immunoreactive
8	Synaptophysin	Dako, 1:100 for 30 mts	HeatPH 6.0 for 35 mts, 1:200 for 30 mts	Non-immunoreactive
9	CD56	Novocastra, 186, 1:100 for 45 mts	Heat DAKO, PH 6.0 for 30 mts, cool 20 mts	Immunoreactive

**Figure 3:**
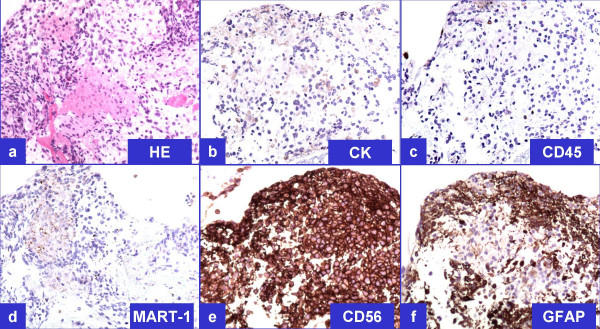
Representative immunoprofile of tumor cells on cellblock sections (see also table 1) **a. **H&E; **b**. Cytokeratin (non-immunoreactive); **c. **LCA (non-immunoreactive); **d. **MART-1 (non-immunoreactive); **e. **CD56 (immunoreactive); **f. **GFAP (immunoreactive).

The tumor cells were non-immunoreactive for cytokeratin 7, cytokeratin 20, cocktail of cytokeratin AE1/AE3 – Cam 5.2, thyroid transcription factor (TTF-1), leucocyte common antigen (LCA, CD45), and MART-1. They were also non-immunoreactive for neuroendocrine immunomarkers, chromogranin and synaptophysin. Non-immunoreactivity for cytokeratin (Figure-3b) ruled out poorly differentiated carcinoma including lung primary, LCA non-immunoreactivity (Figure-3c) ruled out lymphoma and granuloma, nonimmunoreactivity for chromogranin and synaptophysin ruled out paraganglioma, and MART-1 non-immunoreactivity (Figure-3d) ruled out melanoma.

**Figure 4:**
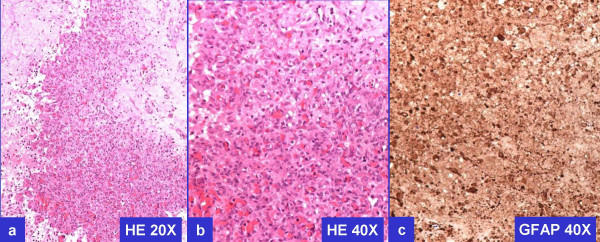
Histomorphology and GFAP immunoreactivity of previously resected GBM.

However, the tumor cells were immunoreactive for CD56 (Figure-3e), consistent with neural differentiation. Based on the past clinical history of recent surgical intervention for recurrent GBM, demonstration of immunoreactivity for GFAP (Figure-3f) confirmed the diagnosis of metastatic GBM. Previously resected GBM demonstrated comparable morphology and immunoprofile (Figure-4)

### Endobronchial biopsy

Endobronchial biopsy was negative for tumor.

### Cytopathology of other material

The bronchoalveolar lavage and pleural fluid cytology were negative for malignant cells.

## Discussion

GBM is by far the most common high-grade glioma representing anaplastic spectrum of fibrillary and diffuse cytoplasmic astrocytoma [2]. It shows marked cellularity, pleomorphism, many mitotic figures, necrosis, and vascular proliferation [2]. The tumor presents usually in the cerebral hemispheres in adults and in brain stem in children. Despite the external-beam radiotherapy and surgery, the median survival for GBM is relatively short (approximately 12 months) [2].

A very few reports describing the cytological features of GBM are found in the literature [9,11,19]. Of the CNS tumors, glioblastoma multiforme is a relatively easy to recognize cytologically as malignant. A review article described cytopathology of various CNS tumors including 15 cases of GBM [19].

However when GBM is sampled from the extra-cranial sites such as lung or lymph node, a differential diagnosis from other tumors may be challenging. Cytologically the smears of GBM show large, spindle to oval, hyperchromatic and pleomorphic nuclei. A fibrillary or necrotic background is also noticed in some of these tumor cells of GBM may be recognizable as astrocytic [1]. But due to the marked pleomorphism of cohesive groups of tumor cells with frequent mitoses, the recognition of GBM from other poorly differentiated tumors may be challenging.

The cell borders of the cohesive tumor cells in the present case were predominantly indistinct with differential diagnosis of poorly differentiated carcinoma, melanoma, paraganglioma, sarcoma, and even lymphoma may be challenging. In the smears with scant cellularity with absence of significant pleomorphism, some groups may resemble granuloma. The smears showing cytoplasmic type of astrocytic tumor cells with well-defined cytoplasm with distinct cell margins may particularly resemble poorly differentiated carcinoma, lymphoma, and melanoma [1].

The tumor cells of GBM are immunoreactive for GFAP and Vimentin [2]. Vimentin immunoreactivity is non-diagnostic due to significant overlap with other lesions in the differential diagnosis of poorly differentiated tumors. Immunoreactivity of tumor cells for GFAP confirms glial differentiation. Generally the astrocytic type of cells shows immunoreactivity for GFAP, but small-undifferentiated cells are often weakly immunoreactive or non-immunoreactive [2]. It is unusual for the gliomas to be positive for epithelial markers such as cytokeratin, thus differentiating them from carcinomas. Although rare, 4% of GBMs may be immunoreactive for cytokeratin, but this is usually weak and focal [2].

Gliosarcoma is other rare subtype of GBM, which shows a characteristic biphasic appearance, consisting of GBM component admixed with sarcomatous elements [17,18]. Extra-cranial metastases of gliosarcoma with sarcomatoid component have been reported in 17 cases (mostly imprint smears with one case as fine needle aspiration biopsy cytology; 7 cases in lung, 6 cases in liver, 2 cases in lymph nodes, 1 in adrenal gland, and 1 in vertebral body) [18].

Although rare, extra-cranial metastasis of GBM may be observed after craniotomy and trephination procedures for initial GBM or more frequently after tumor debulking surgery for a recurrent tumor. A case of metastatic seeding along the needle biopsy tract of a GBM has been reported [16]. Such metastases are attributed to the infiltration of tumor cells into surrounding tissue and extra cranial blood vessels.

Presentation of extracranial metastasis of GBM as a single mass in lung may suggest a differential diagnosis of a poorly differentiated carcinoma [9]. Small cell carcinoma cytologically shows cohesive groups of hyperchromatic, usually small cells, with scant cytoplasm without significant pleomorphism. Rare case reports of a metastatic glioblastoma to cervical lymph node resembling small cell carcinoma have been described in literature [9,11]. In the present case, the small cell carcinoma was not a significant differential diagnosis, as it demonstrated pleomorphism with dusty chromatin and small but visible nucleoli with possibility of poorly differentiated non-small cell carcinoma. Mitoses and apoptotic cells are frequent in small cell carcinoma with many fragile nuclei disrupted as strings of DNA material in the background. The nuclei may be round to oval with 'salt and pepper' chromatin without perceptible nucleoli. Nuclear molding with zig-saw puzzle-like accommodation of nuclear shapes with adjacent nuclei may be present due to scant cytoplasm and delicate nuclear membranes. The tumor cells in this case were non-immunoreactive for cytokeratins and neuroendocrine markers such as chromogranin and synaptophysin, further ruling out the possibility of poorly differentiated carcinoma of lung, both small cell and non-small cell type.

The collection of epitheliod histiocytes in a granuloma show elongated oval carrot shaped nuclei, which usually are not hyperchromatic. The indistinct cell borders impart syncitial appearance to the group. These epitheliod cells are usually admixed with small lymphocytes and may be associated with multinucleated giant cells. In the present case, a few groups of cells did not show pleomorphism but showed occasional lymphocyte admixed with the tumor cells demonstrating ill-defined cell borders superficially resembling a granuloma (Figure-2). In the present case, the granuloma could be ruled out even morphologically, as the smears were relatively cellular with significant proportion of tumor cells showing hyperchromatic and pleomorphic nuclei. In addition, the cells were non-immunoreactive for LCA, which is usually immunoexpressed in epitheloid cells of granuloma.

A paraganglioma shows tumor cells with ill-defined cell borders and nuclei with small nearly visible red nucleoli. The tumor cells may show pleomorphism. The cytoplasm is wispy and finely granular with frayed cell margins. Presence of bare nuclei is frequent [3]. Zellballen arrangement may be observed in cellblock sections. Although, some of the cytomorphological features overlapped, the tumor cells in this case were non-immunoreactive for neuroendocrine markers such as chromogranin and synaptophysin in cellblock sections without Zellballen arrangement excluding paraganglioma.

Individual morphological features of melanoma including prominence of nucleoli, nuclear pseudoinclusions, cytoplasmic melanin pigment, and frequent binucleation with 'demon-eye' nuclei were absent in the present tumor. As melanoma is a great mimicker with diverse morphological patterns, it was ruled out with non-immunoreactivity of these tumor cells for melanoma marker such as, MART-1.

The cells in high-grade large cell lymphomas may show heterogenous population of medium to large pleomorphic non-cohesive cells with clumped nuclear chromatin. The cell borders of singly scattered lymphoma cells are usually distinct [3]. The tumor cells in the present case showed powdery to clear chromatin with cohesive groups of cells without distinct cell borders. They were non-immunoreactive for LCA further ruling out lymphoma.

## Conclusion

Due to the rarity of event, in the absence of proper clinical correlation, extracranial metastases of GBM may be misdiagnosed. In the present case, although the cytomorphological features resembled poorly differentiated non-small cell carcinoma of lung, they were not convincing enough to suggest a specific diagnosis. Further evaluation, including clinical history of previously resected GBM with recurrence and immunophenotyping with appropriate immuno panel, avoided the pitfall leading to proper interpretation of the lung mass in this case as metastatic GBM.

## List of abbreviations

BNCU, Bi chloroethylnitrosourea, (Chemotherapy drug); CNS, central nervous system; FNAB, Fine needle aspiration biopsy; GBM, Glioblastoma multiforme; GFAP, glial fibrillary acidic protein; LCA, leucocyte common antigen; RUL, right upper lobe of lung.

## Authors' contributions

1. (MC) Cytology fellow of the case, collected all the data, participated in cytological evaluation and drafting of manuscript.

2. (HD) Pulmonary fellow of the case and reviewed the manuscript.

3. (JB) Pulmonologist of the case and reviewed the manuscript.

4. (HK) Neuro-oncologist of the case and reviewed the manuscript.

5. (GS) Neuro-surgeon of the case and reviewed the manuscript.

6. (VS) Cytologist of the case, conceptual organization and manuscript writing.

## Consent

This case report is published after consent by the spouse of the deceased patient.
